# Norovirus Genogroup II Epidemics and the Potential Effect of Climate Change on Norovirus Transmission in Taiwan

**DOI:** 10.3390/v14030641

**Published:** 2022-03-20

**Authors:** Shu-Chun Chiu, Szu-Chieh Hu, Ling-Min Liao, Yu-Hua Chen, Jih-Hui Lin

**Affiliations:** Center for Diagnostics and Vaccine Development, Centers for Disease Control, Taipei 11561, Taiwan; schiu@cdc.gov.tw (S.-C.C.); hsc9459wr@cdc.gov.tw (S.-C.H.); lingmin@cdc.gov.tw (L.-M.L.); yuhua1688@cdc.gov.tw (Y.-H.C.)

**Keywords:** norovirus, climate change, epidemiology, Taiwan

## Abstract

The activity of norovirus varies from season to season, and the effect of climate change on the incidence of norovirus outbreaks is a widely recognized yet poorly understood phenomenon. Investigation of the possible association between climatic factors and the incidence of norovirus is key to a better understanding of the epidemiology of norovirus and early prediction of norovirus outbreaks. In this study, clinical stool samples from acute gastroenteritis outbreaks were collected from January 2015 to June 2019 in Taiwan. Data analysis from our study indicated that more than half of the cases were reported in the winter and spring seasons, including those caused by norovirus of genotypes GII (genogroup II).2, GII.3, GII.6, and GII.17, and 45.1% of the patients who tested positive for norovirus were infected by the GII.4 norovirus in autumn. However, GII.6 norovirus accounted for a higher proportion of the cases reported in summer than any other strain. Temperature is a crucial factor influencing patterns of epidemic outbreaks caused by distinct genotypes of norovirus. The results of this study may help experts predict and issue early public warnings of norovirus transmission and understand the effect of climate change on norovirus outbreaks caused by different genotypes and occurring in different locations.

## 1. Introduction

Noroviruses are a major health burden and a leading cause of outbreaks of acute gastroenteritis (AGE) worldwide. Although norovirus gastroenteritis tends to be short-lived and is resolved without medical intervention in otherwise healthy individuals, infections may be more severe among vulnerable populations [1]. The incidence rates of norovirus outbreaks and endemic cases have also increased in Taiwan since 2002 [2]. Noroviruses are highly infectious, largely as a result of their tendency to cause diarrhea among all age groups and their status as foodborne pathogens [3,4,5]. The rate of norovirus-associated hospitalizations in Taiwan was approximately 6.7 per 10,000 person-years [6].

The activities of many of the most common infectious diseases are associated with seasonal patterns and climatic sensitivities, and the determination of these changes in climate or weather conditions may influence the spread of such diseases by affecting the pathogens, which is a major concern [7]. Like other respiratory and gastrointestinal viruses, noroviruses exhibit winter seasonality in temperate climates [8,9,10]. Previous studies have shown that norovirus outbreaks exhibit seasonal patterns with peaks typically in the winter season [11,12,13]. Climate change may modulate the epidemiological outcomes and morbidity and mortality rates of infectious diseases [14,15]. Therefore, if the occurrence of norovirus outbreaks is attributable to weather fluctuations, climate change may influence the incidence and spread of infection. The geographic location of Taiwan provides an ideal environment for viral migration between the tropical and temperate regions. The temporal and spatial variance in norovirus genetic distribution constitutes a significant obstacle to the development of norovirus vaccines and to the early prediction of norovirus outbreaks, for which more extensive and timely surveillance is beneficial [16]. In this study, we investigated the possible association between climatic factors and the incidence of norovirus to encourage a better understanding and early alert to the spread of norovirus infections by monitoring the circulation of epidemic strains in Taiwan.

## 2. Materials and Methods

### 2.1. Sample Collection

Gastroenteritis outbreaks were defined as including two or more cases of gastroenteritis linked in place and time. A new outbreak was arbitrarily defined as an outbreak occurring at least seven days after the last case was reported in a previous outbreak or as an outbreak occurring in a different patient care unit such as a ward or hospital [17,18]. Stool samples from 1811 acute gastroenteritis outbreaks were collected from January 2015 to June 2019 in Taiwan. Acute gastroenteritis cases were defined as cases involving vomiting or diarrhea (three or more loose or liquid stools per day). The biological materials in this study were used for standard diagnostic procedures according to a physician’s prescriptions. We did not conduct sampling in accordance with any specific sampling protocol nor any modification of an established sampling protocol. Following local regulations, this did not require specific consent from patients.

### 2.2. Detection of Norovirus

The specimens from patients were collected and submitted to the Taiwan Centers for Disease Control (TWCDC) for bacterial and viral tests. Bacterial examinations included cultures for common enteric bacteria, namely *Salmonella*, *Shigella*, *Vibrio cholerae*, *Vibrio parahaemolyticus*, pathogenic *E. coli*, *Staphylococcus aureus*, and *Bacillus cereus*; viral tests included real-time reverse transcription polymerase chain reaction (rRT-PCR) tests for norovirus and rotavirus as previously described [19,20,21]. All of the norovirus-positive samples in the rRT-PCR tests were PCR amplified at the open reading frame (ORF) 1/ORF2 junction and were sequenced with primers as previously described [22]. Genotypes were assigned using the Norovirus Genotyping Tool Version 2.0 [23] and the Human Calicivirus Typing Tool [24].

### 2.3. Climate Data Sources

The daily rainfall and daily average values of other meteorological variables during the study period, including the daily maximum temperature, minimum temperature, and duration of insolation, were retrieved from the Taiwan Central Weather Bureau and the Taiwan Climate Change Projection Information and Adaptation Knowledge Platform (TCCIP https://tccip.ncdr.nat.gov.tw/ (accessed on 7 May 2019)).

### 2.4. Statistical Analysis

Data in this study were analyzed using chi square test (*χ*^2^ test), one-way analysis of variance (ANOVA), Kruskal–Wallis test, and linear regression. All tests were two-sided and a *p*-value less than 0.05 was considered to be statistically significant.

## 3. Results

### 3.1. Surveillance of Norovirus Genogroup II

According to the Communicable Disease Control Act, all suspected gastroenteritis outbreaks must be reported to the Taiwan CDC through the Notifiable Diseases Surveillance System and the stool samples have to be collected and sent. A total of 1201 stool samples were collected from patients who were infected with norovirus at any point between 2015 and 2019 in Taiwan and identified nine genotypes of norovirus genogroup II (GII). GII.2 was the most common GII genotype (53.5%) among the samples, followed by GII.4 (13.7%), GII.17 (11.0%), GII.6 (9.2%), GII.3 (7.2%), and others (5.4%, namely GII.5, GII.7, and GII.13). The median age of the patients infected with GII.17 was significantly higher than that of the patients who were infected with the other GII genotypes (median age = 31.1 years, *p* < 0.0001). Furthermore, the median age of the patients infected with GII.3 (7.8 years) was significantly lower than those of the patients infected with the other genotypes (*p* < 0.0001). To further analyze the relationship between age and genotype, we stratified the patients’ ages into six groups: ˂2 years old, 2–5 years old, 5–12 years old, 12–18 years old, 18–64 years old, and ≥65 years old. Results from the age-stratified analysis revealed that GII.3 mainly infected patients in the 5–12-year-old group (50.0%); GII.17 and others mainly infected patients in the 18–64-year-old group (59.1% and 66.2%, respectively). Notably, GII.4 and GII.17 were the most common genotypes in the ≥65 years-old group (18.9% and 13.6%, respectively). In addition, more than half of the patients’ infections were reported in the winter and spring seasons, including those caused by GII.2, GII.3, GII.6, GII.17, and others; GII.6 accounted for a higher proportion of cases reported in the summer (20.7%) than did any of the other genotypes, and this might relate to a higher number of GII.6 [P7] outbreaks occurring in 2019. However, 45.1% of the patients infected in autumn were infected by two different GII.4 epidemic strains, GII.4 [P31] and GII.4 [P16]. Furthermore, GII.2 and GII.3 were mainly identified in student groups (65.6% and 75.6%, respectively), whereas the infections caused by GII.17 and other genotypes were approximately 40% of each in the common patient group. In addition, rates of GII.4 and GII.17 were relatively higher in populous institutes than those of other genotypes (Table 1).

### 3.2. Climate Factors and Geographic Distribution

**Figure 1:**
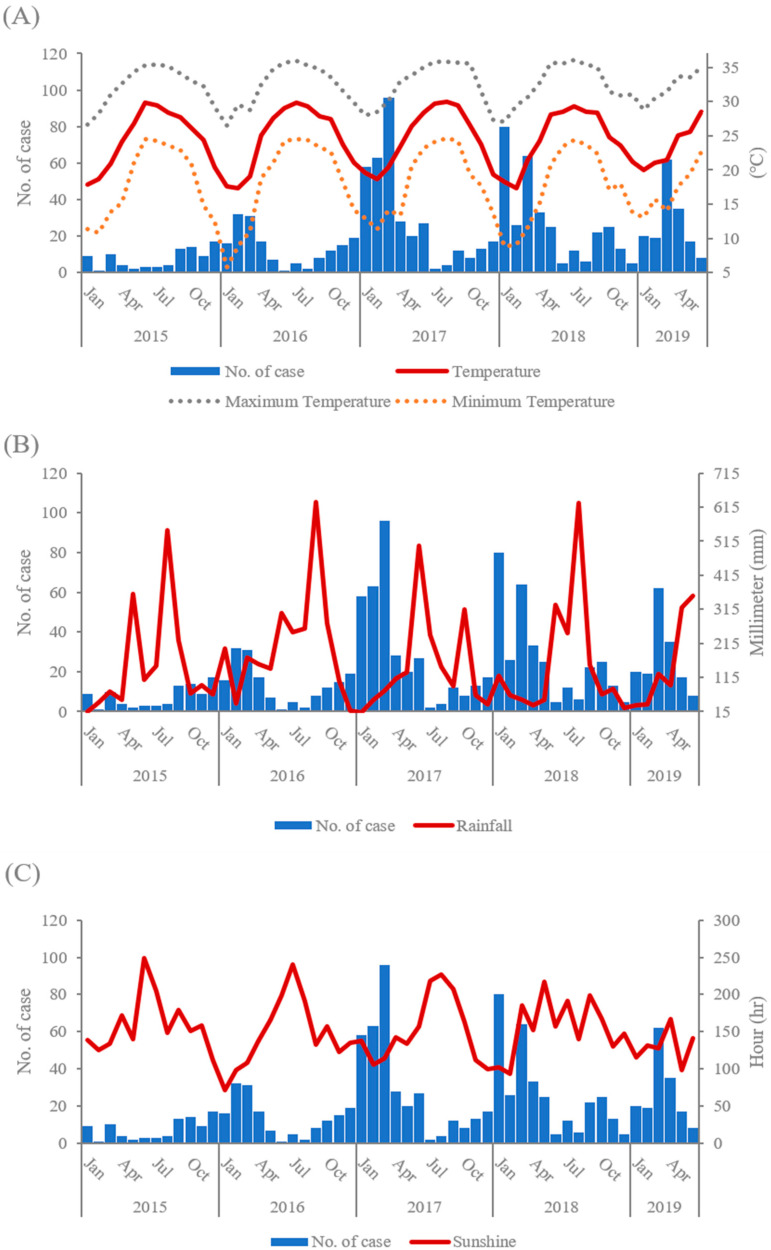
Trends in norovirus infections over time and average daily temperature (**A**), rainfall (**B**), and sunshine (**C**) in Taiwan, 2015–2019.

**Figure 2:**
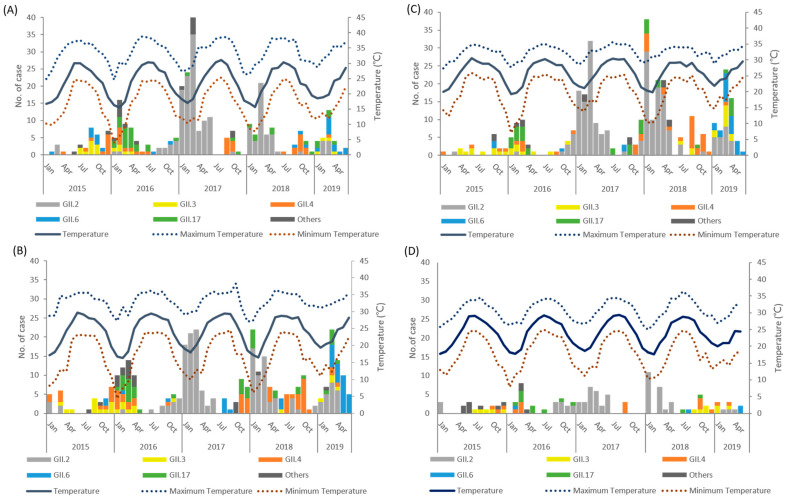
Trends of norovirus genogroup II infections over time and daily temperature in diverse regions of Taiwan, 2015–2019: (**A**) North region; (**B**) West region; (**C**) South region; (**D**) East region. In all regions, except the East region, data archived a level of statistical significance in simple linear regression analysis (*p* < 0.05).

**Figure 3:**
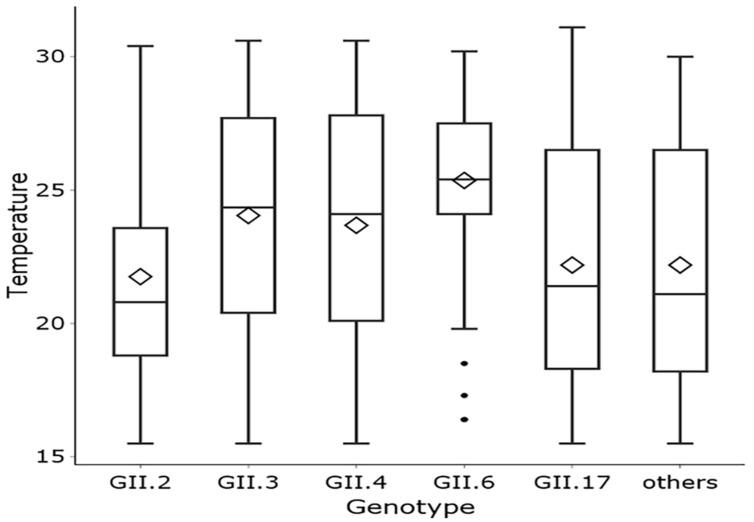
Relationship between norovirus GII genotypes and temperature; *p* < 0.0001 as calculated using the Kruskal–Wallis test.

Taiwan is an island surrounded by seas, and as such, the humidity is relatively high (70–80%) throughout the year. The norovirus outbreaks in Taiwan are less associated with humidity than rainfall. The preliminary analysis indicated a negative correlation between the incidence of norovirus GII and the climatic factors of temperature, rainfall, and sunshine (Figure 1). The monthly confirmed infections of different norovirus GII genotypes in different geographic regions of Taiwan are shown in Figure 2. The geographical distributions of norovirus GII incidence in the north, west, and south regions were similar in regards to the effect of climatic factors. Moreover, we applied multivariate regression to analyze the relationship between incidence of norovirus and climatic factors (Table 2). After an adjustment for rainfall and sunshine, the temperature was found to have a significantly negative correlation with incidence of the virus (β = −0.8773, *p* < 0.0001), which suggests that the spread of norovirus GII gradually decreases as temperature increases. Furthermore, outbreaks of GII.6 norovirus were more likely to occur in the summer than outbreaks of any other genotype, and norovirus GII outbreaks were indeed affected by temperature differences. Therefore, we performed a Kruskal–Wallis test to explore this relationship and created a box plot to display the results (Figure 3). As illustrated by the box plot, outbreaks of GII.6 more often occurred at relatively high temperatures, especially at temperatures higher than 25 °C, compared to outbreaks of other genotypes. Additionally, when the infections were divided among three temperature subgroups: 20–25 °C, 25–30 °C, and 30–35 °C, a significantly higher proportion of patients in the 30–35 °C subgroup (57.1%) were caused by GII.6 than by GII.2 or GII.17 (*p* < 0.05). By contrast, fewer infections in the 20–25 °C group were caused by GII.6 (8.6%) than by any other genotype (*p* < 0.05) (Table 3).

## 4. Discussion

Originally called “winter vomiting disease” [25,26], norovirus has long been associated with cold weather seasonality in temperate climates [8]. The seasonal patterns and climatic sensitivities of various infectious diseases have been studied [27]. However, the relationship between norovirus and weather has not been systematically investigated and the current study is the first detailed attempt to use rigorous statistical methods to demonstrate the association of norovirus infections with climatic, demographic, and viral genotype factors in Taiwan. Using these methods, a greater understanding of the spread of new epidemic strains may be attained, and experts may be able to predict outbreaks of these strains to better protect high-risk groups in data archived statistical significance within the population. Our results indicate that temperature is one of the key factors influencing the occurrence of epidemic outbreaks associated with different genotypes, a trend that is broadly consistent with those explored in previous studies [28,29], and other reports, which have also suggested that rainfall may be play a role in norovirus seasonality [30,31], and thereby suggests that changes in weather patterns could ultimately influence patterns of norovirus epidemics. It is worth noting a finding of significant difference in the age-groups infected by GII.17 and GII.3 viruses from this study as quite relevant, as one of the current models suggest that epidemic GII.4 or GII.17 would infect adults more often than those e.g., GII.2, GII.6, which are more stable overtime [32,33,34].

Norovirus activity in Taiwan similar to that observed in this study, including activity associated with seasonality and temperature differences, has been described previously [35,36]. The observation of GII.4 infections were more prevalent in autumn, and with a double peak occurring between 2018–2019 in Taiwan, this bimodal pattern for seasonality has been reported in Hong Kong during the circulation of three epidemic viruses [37]. However, this study revealed an association between epidemic norovirus GII genotypes and temperature fluctuations that was different from those described in previous reports. These observations suggest that norovirus GII is weather sensitive as a result of the relation to seasonal variation. As is the burden of other infectious diseases [38], norovirus is likely to increase in response to climate change and increasing population density; increasing rainfall and runoff may lead to increased pathogen loads and pathogen survival in the environment. As the majority of norovirus infections are not attributable to contaminated food sources [39], community-level action is likely required to effectively isolate infected individuals, particularly in hotspot areas such as schools and long-term care facilities, to reduce transmission through person-to-person contact.

Further studies that employ larger sample sizes and that cover a greater geographic area are required to validate the consistency of these seasonal trends and to evaluate the potential contribution of other risk factors to norovirus infections. An understanding of how climatic conditions affect the emergence and spread of foodborne diseases is critical for a more comprehensive understanding of current infectious disease dynamics and for prediction of how these dynamics may shift with climate change. Temperature, rainfall, and humidity can influence virus activity, distribution, and pathogenicity [40]. The results of this study may help experts predict norovirus transmission in Taiwan and understand how seasonal changes are likely to influence the location and time of norovirus outbreaks in the long term. This study focused on Taiwanese epidemic strains of norovirus, for which we have extensive data, although we cannot address the origin and genotypic effects introduced from outside the country. However, our analysis revealed specific information regarding norovirus epidemic dynamics; the interplay between co-circulating genotypes; and the interaction between genotypes, demographics, and weather changes. Nevertheless, gene flow and viral spread are global, and new variants can appear anywhere, meaning that global genome sampling is necessary to develop a more complete picture of norovirus transmission. Data from other countries, to be analyzed as thoroughly as the Taiwanese data set was herein, are necessary for the performance of more refined analyses in the future.

In conclusion, the synchronized seasonal patterns and genetic diversity of norovirus observed in Taiwan may inform epidemiological rules for disease prevention and may provide insight into the underlying regular seasonal patterns of norovirus infection. Thus, the continuous monitoring of Taiwanese norovirus outbreaks may contribute to the creation of a complete profile of epidemic patterns that may be used to assist in the early prediction of potential regional norovirus outbreaks.

## Figures and Tables

**Table 1:** Demographic data of patients infected with norovirus by GII genotype in Taiwan, 2015–2019.

Genotype	GII.2 (*n* = 643)	GII.3 (*n* = 86)	GII.4 (*n* = 164)	GII.6 (*n* = 111)	GII.17 (*n* = 132)	Others (*n* = 65)	*p* Value
Age (median, SD)	12.9 (15.0)	7.8 (11.3)	11.5 (30.1)	16.3 (22.6)	31.1 (24.0)	24.0 (17.5)	<0.0001 ^a^
Age group							<0.0001 ^b^
<2 y	4 (0.6)	7 (8.1)	20 (12.2)	1 (0.9)	1 (0.8)	4 (6.2)	
2–5 y	50 (7.8)	12 (14.0)	36 (22.0)	13 (11.7)	4 (3.0)	1 (1.5)	
5–12 y	249 (38.7)	43 (50.0)	28 (17.1)	35 (31.5)	17 (12.9)	15 (23.1)	
12–18 y	88 (13.7)	9 (10.5)	11 (6.7)	10 (9.0)	14 (10.6)	1 (1.5)	
18–64 y	242 (37.6)	15 (17.4)	38 (23.2)	44 (39.6)	78 (59.1)	43 (66.2)	
65+ y	10 (1.6)	0 (0)	31 (18.9)	8 (7.2)	18 (13.6)	1 (1.5)	
Gender							0.3989 ^b^
Male	366 (56.9)	47 (54.7)	88 (53.7)	57 (51.4)	64 (48.5)	31 (47.7)	
Female	277 (43.1)	39 (45.3)	76 (46.3)	54 (48.6)	68 (51.5)	34 (52.3)	
Season							<0.0001 ^b^
Spring	303 (47.1)	27 (31.4)	30 (18.3)	60 (54.1)	58 (43.9)	28 (43.1)	
Summer	38 (5.9)	11 (12.8)	12 (7.3)	23 (20.7)	11 (8.3)	7 (10.8)	
Autumn	31 (4.8)	26 (30.2)	74 (45.1)	20 (18.0)	17 (12.9)	11 (16.9)	
Winter	271 (42.2)	22 (25.6)	48 (29.3)	8 (7.2)	46 (34.9)	19 (29.2)	
Area							0.0439 ^b^
North	176 (27.4)	19 (22.1)	42 (25.8)	30 (27.0)	40 (30.5)	20 (30.8)	
West	177 (27.5)	24 (27.9)	60 (36.8)	39 (35.1)	42 (32.1)	20 (30.8)	
South	225 (35.0)	31 (36.1)	45 (27.6)	39 (35.1)	37 (28.2)	14 (21.5)	
East	65 (10.1)	12 (14.0)	16 (9.8)	3 (2.7)	12 (9.2)	11 (16.9)	
Identity							<0.0001 ^b^
common patient	145 (22.6)	14 (16.3)	29 (17.7)	29 (26.1)	56 (42.4)	28 (43.1)	
Student	420 (65.3)	65 (75.6)	82 (50.0)	59 (53.2)	35 (26.5)	27 (41.5)	
Resident of populous institution	20 (3.1)	3 (3.5)	29 (17.7)	7 (6.3)	19 (14.4)	0 (0)	
Chef/Kitchen worker	17 (2.6)	0 (0)	6 (3.7)	7 (6.3)	5 (3.8)	5 (7.7)	
Nurse	9 (1.4)	2 (2.3)	9 (5.5)	4 (11.1)	9 (6.8)	3 (4.6)	
Prisoner/Military	28 (4.4)	0 (0)	1 (0.6)	1 (0.9)	7 (5.3)	0 (0)	
Others	4 (0.6)	2 (2.3)	8 (4.9)	4 (11.1)	1 (0.8)	2 (3.1)	

Note: Data are presented as *n* (%), SD = standard deviation. ^a^ *p* value was calculated using the Kruskal-Wallis test. ^b^ *p* value was calculated using the *χ*^2^ test.

**Table 2:** Relationship between norovirus and climatic factors in Taiwan, 2015–2019.

Multivariate Linear Regression Model			
Variate	Estimate parameter	*p*-value	R^2^
Tempareture	−0.8773	<0.0001	0.1712
Rainfall	0.0014	0.6303	
Sunsine	0.0328	0.0022	

**Table 3:** Relationship between norovirus genogroup II genotypes and temperature in Taiwan, 2015–2019.

Norovirus Genotype	GII.2	GII.3	GII.4	GII.6	GII.17	Others	*p* Value
Average Temperature (°C)							<0.0001
15–20	210 (32.7)	19 (22.6)	36 (22.8)	9 (8.6) ^a^	49 (38.6)	24 (40.0)	
20–25	300 (46.7)	26 (31.0)	61 (38.6)	36 (34.3)	43 (33.9)	11 (18.3)	
25–30	132 (20.6)	39 (46.4)	61 (38.6)	60 (57.1) ^b^	35 (27.6)	25 (41.7)	

Note: Data are presented as *n* (%), SD = standard deviation. ^a^ *p* < 0.05 (*χ^2^* test) for GII.6 vs. GII.2. GII.3, GII.4, GII.17 and others. ^b^ *p* < 0.05 (*χ^2^* test) for GII.6 vs. GII.2 and GII.17.

## Data Availability

The data presented in this study are all presented in the manuscript.
